# P-1863. Integrated outpatient treatment of Serious Injection-Related Infections and Opioid Use Disorder: the Buprenorphine plus Outpatient Parenteral Antibiotic Therapy (BOPAT) Clinical Trial

**DOI:** 10.1093/ofid/ofaf695.2032

**Published:** 2026-01-11

**Authors:** Laura Fanucchi, Alice C Thornton, Evelyn Villacorta Cari, Hilary L Surratt, Paul Nuzzo, Sharon Walsh, Connor VanMeter, Sean Murphy, Shashi Kapadia, Michelle Lofwall

**Affiliations:** University of Kentucky, Lexington, Kentucky; The University of Kentucky, Lexington, Kentucky; University of Kentucky, Lexington, Kentucky; University of Kentucky, Lexington, Kentucky; University of Kentucky, Lexington, Kentucky; University of Kentucky, Lexington, Kentucky; University of Kentucky, Lexington, Kentucky; Weill Cornell Medical College, New York, New York; Weill Cornell Medical Center, New York, New York; University of Kentucky, Lexington, Kentucky

## Abstract

**Background:**

Outpatient parenteral antibiotic therapy (OPAT) is often denied to persons with opioid use disorder (OUD) and serious injection-related infections (SIRI) despite increasing evidence that it may be feasible and safe. *Staphylococcus aureus* (SA) causes the majority of SIRI, and SA infections have high morbidity. This study evaluates the efficacy and cost-effectiveness of an integrated outpatient care model combining Buprenorphine for OUD with OPAT (BOPAT; NCT04677114) compared to Treatment As Usual (TAU). The aim is to describe baseline characteristics, healthcare utilization, peripherally-inserted central catheter (PICC) complications, and serious adverse events (SAE) with a focus on SA.

**Figure 1.**
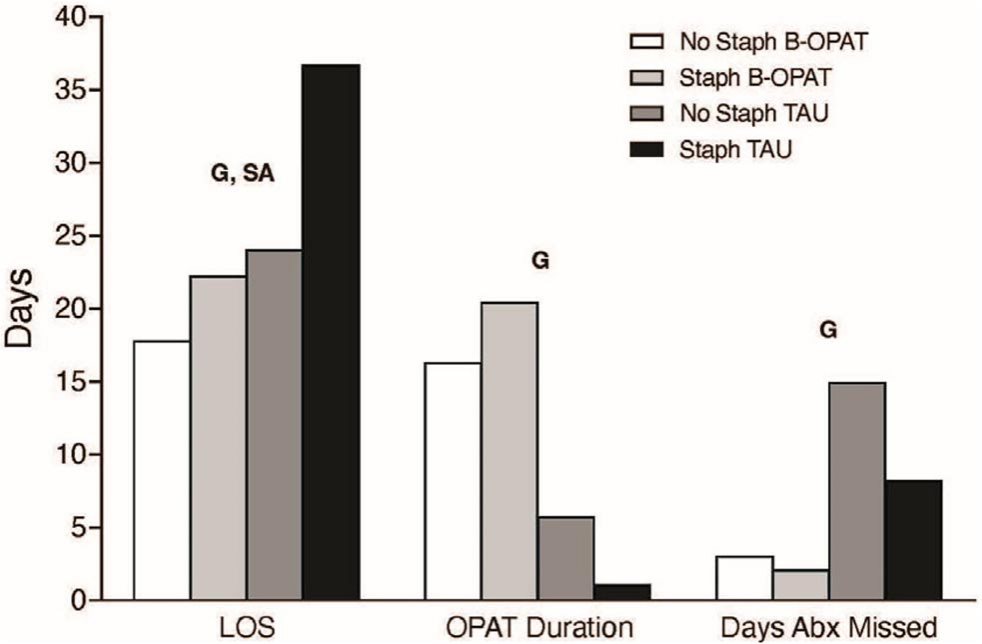
Length of hospital stay (LOS), OPAT duration, and missed days of initially planned IV antibiotic course in each group, stratified by whether the participant had an infection with Staphylococcus aureus. ANOVA was completed for each outcome testing for main effects of Group (B-OPAT, TAU) and Staphylococcus aureus (SA: yes/no) and Group X SA interaction. G indicates significant main effect of group, and SA indicates significant main effect of SA (p<0.5) displayed above each outcome. There were no significant Group x SA interactions.

**Figure:**
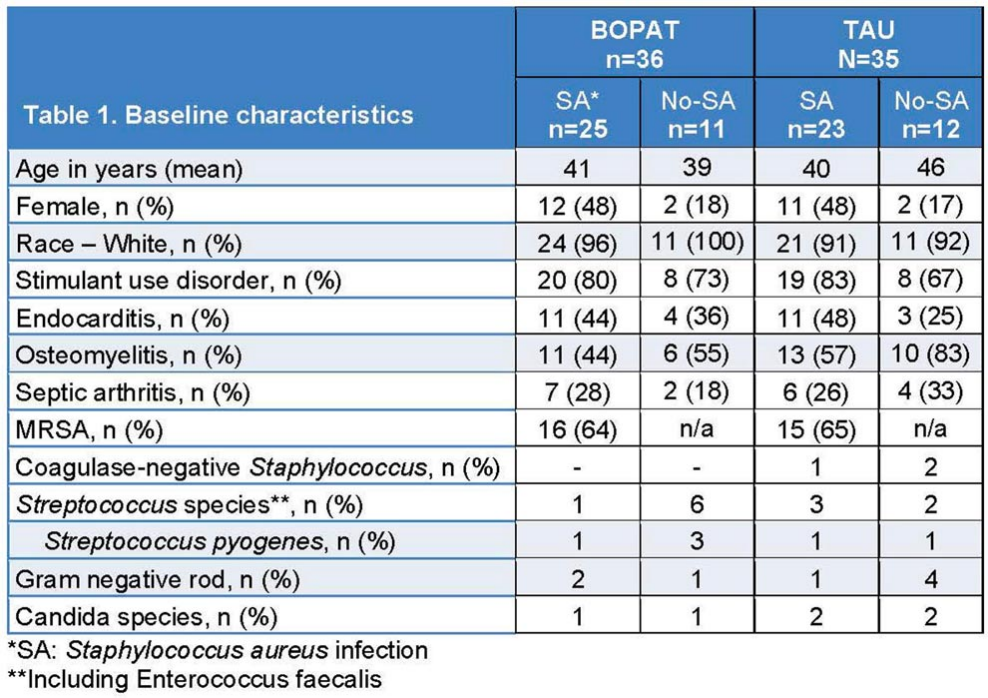

**Methods:**

BOPAT was a randomized, 2-arm superiority trial in hospitalized adults with OUD and SIRI. Participants were randomized 1:1 to discharge with BOPAT or TAU. Healthcare utilization, antibiotic therapy, PICC complications, and AEs are collected at baseline and through the 12-week post-discharge intervention.

**Figure:**
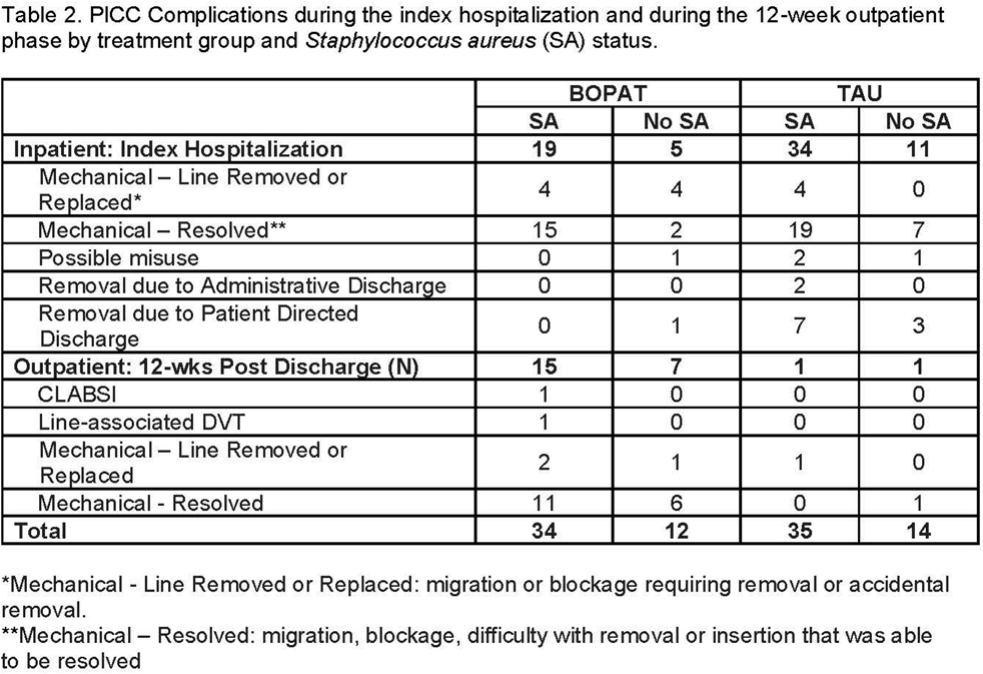

**Results:**

From March 2021 – March 2025, 71 people were randomized (BOPAT: 36; TAU: 35), with 48 (68%) having SA (BOPAT: 25; TAU: 23), with 31 (65%) due to Methicillin-resistant SA. Baseline characteristics stratified by SA are shown in Table 1. Average hospital length of stay in BOPAT: 20.9 ±8.9 days vs. TAU: 32.4 ±16.0 (p=0.001). Though participants with SA had longer LOS there was no significant interaction between group and SA (p=0.200). BOPAT participants completed 19.2 ±10.0 days of OPAT vs. 2.7 ±8.3 in TAU (p< 0.001) and BOPAT participants missed 2.4 ±4.6 planned antibiotic days vs. 10.6 ±13.8 in TAU (p=0.001) (Figure 1). Over 12-weeks post-discharge, 14 (39%) BOPAT participants had SAEs, including 9 readmissions related to SIRI and 1 death. In TAU, 20 (57%) participants had SAEs, including 15 readmissions related to SIRI, and 3 deaths. Absolute number of PICC complications were similar between groups with more happening in those with SA (Table 2).

**Conclusion:**

BOPAT is an innovative outpatient care model that reduces hospital length of stay, improves completion of planned antibiotic therapy, and has improved safety outcomes compared to TAU. These results are particularly impactful as they challenge the common assumption that OPAT is not safe in persons who inject drugs.

**Disclosures:**

Sharon Walsh, PhD, Astra Zeneca: Advisor/Consultant|Braeburn Pharmaceuticals: Advisor/Consultant|Cerevel Therapeutics: Advisor/Consultant|Indivior: Advisor/Consultant|Reacx: Advisor/Consultant Michelle Lofwall, MD, Braeburn Pharmaceuticals: Advisor/Consultant|Camurus: Honoraria

